# Supplementary material for: What Has Been the Impact of Covid-19 on Safety Culture? A Case Study from a Large Metropolitan Healthcare Trust
Source: Int J Environ Res Public Health. 2020 Sep 25;17(19):7034. doi: 10.3390/ijerph17197034 (PMC7579589; doi:10.3390/ijerph17197034)
Supplement: Supplementary file 1 [file ijerph-17-07034-s001.pdf]

APPENDIX A. BASELINE QUESTIONNAIRE

About you

1.1 How many years of experience do you have working in Imperial College Healthcare NHS Trust?

1.2 What type of contract do you have with the trust?

1.3 Which of the following best describes your role or profession?

1.4 Which site do you spend most time at?

1.5 What division do you work in?

1.6 Directorate: (Corporate)

1.7 Directorate: (Medicine & Integrated Care)

1.8 Directorate:(Surgery, Cancer & Cardiovascular Sciences)

1.9 Directorate: (Woman's, Children's, and Clinical Support)

Safety Attitudes Questionnaire

2.1 Where I work, it is difficult to speak up if I perceive a problem with patient care

2.2 Disagreements are resolved appropriately where I work (i.e. not who is right, but what is best for the patient)

2.3 It is easy for staff here to ask questions when there is something that they do not understand

2.4 The staff here work together as a well-coordinated team

2.5 I would feel safe being treated here as a patient

2.6 Clinical errors are handled appropriately where I work

2.7 I know the proper channels to direct questions regarding patient safety where I work

2.8 I receive appropriate feedback about my performance

2.9 It is difficult to discuss errors where I work

2.10 I am encouraged by my colleagues to report any patient safety concerns I may have

2.11 The culture where I work makes it easy to learn from the errors of others

2.12 My suggestions about safety would be acted upon if I expressed them to my line manager (Your line manager is the manager you report directly to. Typically this manager will conduct your performance appraisal)

2.13 I like my job

2.14 This is a good place to work

2.15 I am proud to work where I do

2.16 Morale is high where I work

2.17 When my workload becomes excessive, my performance is impaired

2.18 I am less effective at work when fatigued

2.19 I am more likely to make errors in tense or hostile situations

2.20 My line manager supports my daily effortsYour line manager is the manager you report directly to. Typically this manager will conduct your performance appraisal

2.21 Senior leaders don't knowingly compromise patient safetySenior leaders in this context refers to our organisation's executive team, corporate directors, (i.e. medical director, nurse director, director of people and OD etc.) and divisional board including divisional directors, divisional directors of operations, divisional directors of nursing

2.22 Senior leaders are doing a good jobSenior leaders in this context refers to our organisation's executive team, corporate directors, (i.e. medical director, nurse director, director of people and OD etc.) and divisional board including divisional directors, divisional directors of operations, divisional directors of nursing

2.23 Problem staff are dealt with constructively in our organisation

2.24 I get adequate & timely information about events that might affect my work, from my line managerYour line manager is the manager you report directly to. Typically this manager will conduct your performance appraisal

2.25 Where I work the levels of staffing are sufficient to handle the number of patients

2.26 This organisation does a good job of training new staff

2.27 Trainees/Students in my area of work are adequately supervised

2.28 I experience good collaboration with nurses where I work

2.29 I experience good collaboration with doctors where I work

2.30 Communication breakdowns that lead to delays in delivery of care are common

2.31 I have support I need from other staff to care for patients

2.32 All the necessary information for diagnostic and therapeutic decisions is routinely available to me

2.33 Fatigue impairs my performance during emergency situations (e.g. emergency resuscitation, seizure)

APPENDIX B. COVID-19 QUESTIONNAIRE

About You

1.1 Age

1.2 Gender

1.3 Ethnicity - British census category (Optional)

1.4 Role

1.5 Where are you based?

1.6 Specialty or area that you were working in prior to the COVID pandemic?

1.7 Have you been redeployed to a different area, hospital, or specialty?

1.8 If so, to which specialty or area have you been redeployed?

1.9 If redeployed: how would you describe the training you received in preparation for redeployment?

1.10 If redeployed: how would you describe the support you received during redeployment?

1.11 How many days did you spend at work in the last 7 days?

1.12 What was the average duration of your shift?

1.13 Approximately how many cases of suspected or confirmed COVID-19 are in your hospital or place of work?

1.14 Have you treated a COVID-19 positive patient in the past week?

1.15 In the past week, what is your own COVID-19 status?

1.16 I work in a

1.17 (Optional) Current Hospital/Trust

Safety Attitudes Questionnaire

2.1 Nurse input is well received in this clinical area.

2.2 In this clinical area, it is difficult to speak up if I perceive a problem with patient care.

2.3 Disagreements in this clinical area are resolved appropriately (i.e., not who is right, but what is best for the patient).

2.4 I have the support I need from other personnel to care for patients.

2.5 It is easy for personnel here to ask questions when there is something that they do not understand.

2.6 The physicians and nurses here work together as a well-coordinated team.

2.7 I would feel safe being treated here as a patient.

2.8 Medical errors are handled appropriately in this clinical area.

2.9 I know the proper channels to direct questions regarding patient safety in this clinical area.

2.10 I receive appropriate feedback about my performance.

2.11 In this clinical area, it is difficult to discuss errors.

2.12 I am encouraged by my colleagues to report any patient safety concerns I may have.

2.13 The culture in this clinical area makes it easy to learn from the errors of others.

2.14 My suggestions about safety would be acted upon if I expressed them to management.

2.15 I like my job.

2.16 Working here is like being part of a large family.

2.17 This is a good place to work.

2.18 I am proud to work in this clinical area.

2.19 Morale in this clinical area is high.

2.20 When my workload becomes excessive, my performance is impaired.

2.21 I am less effective at work when fatigued.

2.22 I am more likely to make errors in tense or hostile situations.

2.23 Fatigue impairs my performance during emergency situations (e.g. emergency resuscitation, seizure).

2.24 Management supports my daily efforts.

2.25 Management doesn’t knowingly compromise patient safety.

2.26 Management is doing a good job.

2.27 Problem personnel are dealt with constructively by our management.

2.28 I get adequate, timely info about events that might affect my work, from management.

2.29 The levels of staffing in this clinical area are sufficient to handle the number of patients.

2.30 This hospital does a good job of training new personnel.

2.31 All the necessary information for diagnostic and therapeutic decisions is routinely available to me.

2.32 Trainees in my discipline are adequately supervised.

2.33 I experience good collaboration with nurses in this clinical area.

2.34 I experience good collaboration with staff physicians in this clinical area.

2.35 I experience good collaboration with pharmacists in this clinical area.

2.36 Communication breakdowns that lead to delays in delivery of care are common.

Oldenburg Burnout Inventory

3.1 I always find new and interesting aspects of my work.

3.2 There are days when I feel tired before I arrive at work.

3.3 It happens more and more often that I talk about my work in a negative way.

3.4 After work, I tend to need more time than in the past in order to relax and feel better

3.5 I can tolerate the pressure of my work very well.

3.6 Lately, I tend to think less at work and do my job almost mechanically.

3.7 I find my work to be a positive challenge.

3.8 During my work, I often feel emotionally drained.

3.9 Over time, one can become disconnected from this type of work.

3.10 After working, I have enough energy for my leisure activities.

3.11 Sometimes I feel sickened by my work tasks.

3.12 After my work, I usually feel worn out and weary.

3.13 This is the only type of work that I can imagine myself doing.

3.14 Usually, I can manage the amount of my work well.

3.15 I feel more and more engaged in my work.

3.16 When I work, I usually feel energized.

Hospital anxiety and depression scale

4.1 I feel tense or 'wound up'

4.2 I still enjoy the things I used to enjoy

4.3 I get a sort of frightened feeling as if something awful is about to happen

4.4 I can laugh and see the funny side of things

4.5 Worrying thoughts go through my mind

4.6 I feel cheerful

4.7 I can sit at ease and feel relaxed

4.8 I feel as if I am slowed down

4.9 I get a sort of frightened feeling like 'butterflies' in the stomach

4.10 I have lost interest in my appearance

4.11 I feel restless as I have to be on the move

4.12 I look forward with enjoyment to things

4.13 I get sudden feelings of panic

4.14 I can enjoy a good book or radio or TV program

Any other comments

5.1 Is there anything else you would like to add or comment on, especially in relation to redeployment, wellbeing, or safety during Covid-19?

APPENDIX C. SAQ SCORING

Scale	2017 Questions	2020 Questions
Teamwork climate	1*,2,31,3,4	1§,2*,3,4,5,6
Safety Climate	5,6,7,8,9,10,11*	7,8,9,10,11*,12,13
Job satisfaction	13,14,15,16	15,16§,17,18,19
Stress recognition	17,18,19,33	20,21,22,23
Perceptions of management	20,21,22,23,24	24,25,26,27,28
Working conditions	25,26,32,27	29,30,31,32
Overall	All subscale questions, 12, 28,29,30*	All subscale questions, 14, 33, 34, 35§, 36*

*negatively scored, §excluded for the purposes of matching
